# Combined effects of Telehealth and Modified Constraint-Induced Movement Therapy for Individuals with Chronic Hemiparesis

**DOI:** 10.5195/ijt.2020.6300

**Published:** 2020-06-30

**Authors:** Mary Ann Smith, Machiko R. Tomita

**Affiliations:** 1 University of St. Augustine for Health Sciences, Austin, TX, USA; 2 University at Buffalo, Buffalo, NY, USA

**Keywords:** mCIMT, Hemiparesis, Telehealth

## Abstract

Telehealth use allows improved access to services and results in potential cost savings. The purpose of this study was to examine the effectiveness of a combined modified Constrained Induced Movement Therapy (mCIMT) program using telehealth and in-person sessions, for participants with higher (Group 1) and lower (Group 2) functional ability of the hemiparetic upper extremity. Using a pre-experimental design with a 6-week intervention, 28 participants were assessed twice on use of upper extremity via subjective and objective measures. For the Motor Activity Log, the amount of use and quality of use were significant for Groups 1 and 2. Significant improvements were shown on the Wolf Motor Function Test (WMFT), the Fugl-Meyer UE, and the Functional Independence Measure (FIM) for both groups except for the strength subtest on the WMFT and the timed portion for Group 1. Percentages of attendance for telehealth and in-person sessions were also compared. Telehealth sessions had a higher attendance rate (84.5%) than in-person sessions (75.3%) (p=.004). The combined mCIMT program of telerehabilitation and in-person group sessions was effective in improving functional ability after a stroke.

Stroke is a prevalent cause of disability and death throughout the world. Damage to the nervous system after a person experiences a stroke often results in hemiparesis, affecting use of the arm and hand on the hemiparetic side. Approximately 80% of individuals with stroke experience difficulties in performing activities of daily living (ADLs); consequently, social participation is limited because of the impairments (Kwakkel et al., 2015). Research exploring the effects of deafferentation in primates led to the discovery of the phenomenon of “learned non-use,” in which an individual avoids the use of the affected limb due to pain, aversion, or repeated failure from previous attempts (Taub et al., 1994). This leads to the predominant use of the unaffected limb as a compensatory technique to overcome the lack of mobility in the affected upper limb (Nijland et al., 2013). The approach of Taub et al. (1994), constraining the non-affected limb and forcing use of the affected limb is termed Constraint Induced Movement Therapy (CIMT). Preliminary research on primates was found to improve functional use of the affected upper extremity (UE) and was foundational in addressing “learned non-use.” CIMT has been shown to reverse the phenomenon of “learned non-use” in individuals who experienced a stroke (Kwakkel et al., 2015, Taub et al., 2005; Wolf et al., 2008; Wen et al., 2014).

Traditional CIMT focuses on restraining the unaffected UE for up to 18 hours per day in order to force the affected UE to be used during both functional and strengthening tasks (Nilsen et al., 2015; Treger et al., 2012). While the unaffected UE is restrained, participants of traditional CIMT partake in intensive therapy that requires use of only the affected UE. By focusing on using the affected UE, traditional CIMT can combat the learned non-use phenomenon and helps the participant begin to use their affected UE in a functional way (Wu et al., 2007).

Although CIMT has been shown to be an effective intervention, the intensity of the approach requires high participant adherence. According to Schaumberg et al. (1999), (conference paper cited by Page et al., 2004), there is only a 32% compliance rate with the traditional CIMT protocol. Several reasons for this low compliance rate may be the impracticality of the time the affected UE is restricted, the intensive amount of therapy, or participants' lack of adherence to the protocol (Page et al., 2004). Other limitations with CIMT include concerns about cost effectiveness, worthiness of the cost, and concerns about cost reimbursement (Shi et al., 2011).

Modified CIMT (mCIMT) protocol involves a family of treatments (Taub et al., 1999). The restraint procedures are altered, and the amount of therapy is distributed over a longer period (Page et al., 2004). For example, the study conducted by Yu et al. (2017) regarding the effect of mCIMT on subcortical cerebral infarction only required the participants to wear a mitt on their unaffected arm for 30% of their waking hours, whereas the studies by Nijand et al. (2012), El-Helow et al. (2015), and Smania et al. (2012) mandated that their participants wear the constraining mitt for a minimum of 3 hours, 6 hours, and 12 hours a day, respectively. Variation in intensity level, length of time of constraint, and length of mCIMT program resulted in improved tolerance and increased compliance with many participants (El-Helow et al., 2015; Yu et al., 2017).

A newer approach is Internet-based constraint-induced movement therapy (iCIMT); iCIMT is a mCIMT approach that incorporates telehealth. Telehealth allows delivery of therapy services to individuals who may not have the ability to frequently access the clinic, as both mCIMT and CIMT approaches require consistent adherence for therapy sessions (AOTA 2018; Jacobs et al., 2015). The rise of telehealth allows for therapy services to be provided through technology, including telephones, videoconferencing, personal digital assistants, smartphones, and virtual reality (McCue et al., 2010). Technology-based rehabilitation services may benefit individuals who have difficulty traveling to a clinic setting, those in rural areas, or even patients with chronic conditions that require frequent visitation (AOTA 2018; Jacobs et al., 2015). This is a client-centered approach, providing increased access to CIMT and mCIMT programs.

Telehealth is a fast-growing service delivery model that shows potential to create, implement, and maintain health promoting habits and routines. At the time of this writing, a COVID-19 pandemic waiver allows occupational therapists to be reimbursed for occupational therapy services provided through telehealth to Medicare beneficiaries. However, prior to the COVID-19 pandemic, Medicare did not recognize occupational therapy practitioners as eligible providers of therapy services through telehealth (AOTA 2019). Therefore, it is imperative that clinical research demonstrate the effectiveness of programs for individuals with hemiplegia, incorporating newer technologies and potentially cost-effective interventions.

In most studies using mCIMT, individuals with severe spasticity and/or limited wrist and hand movements were excluded, in accordance with the general guidelines for mCIMT (Taub et al., 1999). However, Wolf (2007) reports improved function in participants with severe hemiparesis, citing case reports; Bonifer & Anderson (2003) and Bowman et al. (2006). Although the case reports have mixed results; Taub et al. (1999) report that a larger percentage of participants with lower function in the hemiparetic UE, qualify for CIMT, and improve in functional abilities, than was previously expected. There are many unanswered questions about improvement in functional ability following CIMT in lower functioning individuals with hemiparesis. In this study we have included participants who are lower functioning in their affected UE. In addition, we encouraged informal caregivers to attend sessions.

The purpose of this study was to examine the effectiveness of a combined approach for a mCIMT program, which is traditional group rehabilitation and iCIMT telehealth technology, on increasing the use of the affected UE in individuals who are both higher and lower functioning. Expanding on previous studies, a combination of iCIMT with the addition of in-person group sessions, may enhance adherence with the CIMT treatment regimen. To our knowledge, this is the first study using two types of CIMT to identify its effectiveness with hemiplegia.

## RESEARCH HYPOTHESES

We hypothesize that the combined use of mCIMT and iCIMT with individuals with hemiparesis will

(1) Improve functional use of the upper extremity and hand for higher and lower functioning participants.

(2) Improve subjective report on the amount of use and quality of movement in the hemiparetic UE for daily activities for higher and lower functioning participants and

(3) Improve ADL participation for higher and lower functioning participants

In addition, we hypothesize that

(4) Internet sessions will have greater participant adherence compared to that of the once weekly in-person sessions.

## METHODS

### STUDY DESIGN

This research employed a pre-experimental design with a 6-week intervention period. Group interventions took place in a lab space at our university once per week. iCIMT sessions occurred twice a week, using web conferencing technology in participants' homes. Assessments were conducted twice, at baseline and six weeks later. Both individual and group sessions required sign-in attendance to determine participant adherence to the program.

### PARTICIPANTS

The sample size was determined using a power analysis based on a study by Page et al. (2008). The effect size was Cohen's d = 0.88. With 20 participants we can achieve the power of .80, allowing 5% Type I error. Anticipating 20% of attrition and considering this is a new approach, we recruited 32 participants using a convenience sampling method. Our data analysis included 28 participants. Four participants did not complete the study; two decided to withdraw after pre-testing and two participants could not be assessed at posttest due to extenuating circumstances. The 28 participants were divided into two groups based on their performance on the timed portion of the Wolf Motor Function Test (WMFT) at baseline. Since the WMFT timed portion measures functional ability ranging from gross to fine motor, it was determined that this was the best objective measurement to use to sort the participants into groups. The cutoff point was 38, the mean score. There were 15 higher functioning and 13 lower functioning participants.

Recruitment methods included informational advertisement in local rehabilitation facilities, in-services to stroke support groups, distribution of flyers, phone calls, letters, and emails, to area clinicians. Inclusion criteria were (1) 18-75 years of age, (2) presence of consistent hemiparesis and impaired function, (3) ability to transfer self-independently, (4) at least six months post-stroke, (5) being able to follow two-step written and/or verbal directions, (6) having at least 15 degrees of active shoulder flexion and abduction, (7) having at least 5-10 degrees of active wrist flexion and 5-10 degrees of active wrist extension from neutral, (8) having at least 5-10 degrees of flexion in the thumb and/or digits, and (10) living in their own home with a device capable of video conferencing and Internet access. Participants were excluded from the study if: (1) they were living in a nursing home, (2) they were receiving outside occupational therapy services, and/or (3) they did not understand and/or speak English at fifth grade level.

### PERFORMANCE MEASURES

Use of the hemiparetic upper extremity can be measured through functional use, motor ability, as well as the participant's perception of the use of their affected arm. To assess the quality of movement and strength while performing different tasks, the Wolf Motor Function Test (WMFT) was implemented. The WMFT is a standardized assessment that measures 17 gross and fine motor tasks in the affected UE. The items include motions such as: forearm to the table, extended elbow, hand to the table, and lifting a basket, among others. Each item is timed except for the two strength subtests. Subtests progress from gross motor to complex fine motor movement targeting distal musculature. In addition to timed subtests, raters score participants' functional ability preforming the subtests based on video recordings. The Functional Ability Scale section of the WMFT is based on a 6-point ordinal scale. A score of 0 indicates no use, while a score of 5 indicates “normal” functional ability (Wolf et al., 2001).

Morris et al. (2001) found the test-retest reliability was very high for both the Functional Ability Scale (r = 0.95) and timed subtests (r=0.90) of the WMFT. Nijland et al. (2010) found that Spearman's rank correlation coefficients ranged from 0.70 to 0.86. ICCs for inter-rater and intra-rater reliability ranged from 0.92 to 0.97.

The Motor Activity Log (MAL) assesses functional participation through a standardized structured interview; participants rate the Amount of Use (AOU) and Quality of Use (QOU) of the affected limb on a scale of 0 to 5. A score of 5 indicates the affected limb performs at the same level as before the stroke (Uswatte et al., 2006). The MAL's internal consistency was high (Cronbach's alpha = 0.94), and concurrent validity with the Action Reach Arm Test (ARAT) has been established (Uswatte et al., 2006). It has also excellent test-retest reliability (Van der Lee et al., 2004).

The Fugl-Meyer Assessment of the UE (FMA-UE) contains 33 standardized items that assess motor impairment in the upper extremity post-stroke. FMA-UE items assess movement synergies, isolated movement, hand and wrist function, speed, dexterity, and reflexes, according to a 3-point scale. A score of 0 indicates the individual cannot perform the item, a score of 1, partial performance, and a score of 2, full performance. The overall possible score is 66, which indicates optimal recovery. The FMA has high test-retest reliability (r= 0.834 to 0.972) (Kim et al., 2012).

The Functional Independence Measure (FIM) addresses performance in activities of daily living (ADL). A score of 7 indicates total independence in an ADL, a score of 1 is total dependence, and scores between 2 and 6 indicate the level of assistance needed to complete the task and/or need for adaptive equipment (Ottenbacher et al., 1996). Ottenbacher et al. (1996) reported reliability values across the 11 studies. The results revealed a median interrater reliability for the total FIM of .95 and median test-retest and equivalence reliability values of .95 and .92, respectively. For the purposes of this study, we used the self-care section of the FIM that includes feeding, grooming, bathing, toileting, tub/shower transfers, and dressing. We included the FIM in this study based on its ability to track the abilities of patients with chronic disease (Uniform Data System for Medical Rehabilitation, 2014).

### PROCEDURE

**Figure 1.**
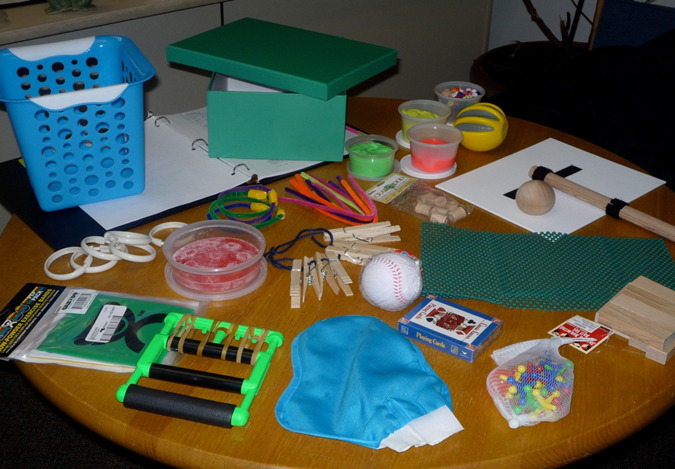
Tool kit for iCIMT participants.

First, we acquired an IRB approval. Then, we conducted telephone screenings to assess participants' eligibility. Written consent was obtained during the pretest, which took place at the university lab site, followed by the administration of the WMFT, FMA, MAL, and FIM. Then, a home visit was made to provide education and set up web-based technology for video conferencing capability. At the time of the home visit, a tool kit containing common objects for gross and fine motor movements was provided to each participant for use at home during the Internet-based sessions. (See Figure 1).

Internet session interventions occurred two times a week; one hour each session. Web-based technology, either Google Hangout or Adobe Connect were utilized. The participant and the therapist met, and the computer screens were adjusted allowing full view of the hemiplegic UE and hand in order to observe participants' engagement in tasks. In-person sessions were conducted once a week in a group setting lasting 1 to 1.5 hours for a total of three sessions per week. For the in-person sessions, social interaction among participants were encouraged in addition to group activities that facilitated engagement in occupation-based tasks. Examples of group sessions included the following: food preparation, games, painting, crafts, seasonal activities, and various simulated instrumental ADL (IADL) tasks.

Both Internet and in-person sessions focused on completing a variety of gross and fine motor exercises to encourage transfer of tasks to participants' ADLs/IADLs. In addition, participants were provided with a log to document adherence to the protocol and were requested to wear the mitt on the unaffected UE for about 4 hours per day. The in-person group session and the Internet sessions addressed daily activities that participants valued encouraging the transfer of motor abilities from therapy sessions to real-life situations. The “transfer package” that this study offered consisted of home skills assignments and discussion and demonstration on using the affected UE in daily activities (Taub, 2012). Throughout the sessions, participants were encouraged to problem solve and improve their task performance though active participation and feedback; incorporating a motor relearning/task-oriented approach into the sessions (Chan et al., 2006). Key components of the motor re-learning/task oriented approach were provided by structuring practice sessions, encouraging engagement in preferred tasks, fitting the task to the individual with just the right challenge, using strategies such as shaping to refine skills, and providing opportunities for intrinsic and extrinsic feedback (Almhdawi et al., 2014; Sabari et al., 2014).

These methods were used to measure adherence to the treatment program and to encourage participants to generalize the results found during treatment sessions to real-world environments. The WMFT, FMA, MAL, and FIM were administered at the end of the 6-week session to assess functional use of the affected UE and hand in daily activities.

### STATISTICAL ANALYSIS

We used a two-tailed independent t-test to compare age and months post stroke, and chi-square for ethnicity, sex, marital status, and affected side for our pretest demographic characteristics. A one-tailed independent t-test compared pretest scores on functional outcome measures between group 1 (higher functioning group, n=15) and group 2 (lower functioning group, n=13). We anticipated that the higher functioning group's pre-test scores would be better than the lower functioning group.

To address the first three research hypotheses, we used a one-tailed paired t-test for the total scores of the WMFT (timed and functional ability scales), FMA-UE, MAL and FIM, for Group 1 and Group 2, separately. In order to control Type I error in multiple measures of eight outcome variables, we used Benjamini-Hochberg method using the false discovery rate (FDR). This method decreases the possibility of the false discovery rate; therefore, it can control the event that small p-values (<.05) happen by chance, leading to incorrectly rejecting the true null hypothesis (Benjamini & Hochberg, 1995). This method uses sequential modified Bonferroni correction for multiple hypothesis testing. Controlling the FDR instead of the Family Wise Error Rate in Bonferroni correction, Benjamini-Hochberg method is less stringent and increases the method's power (Benjamini & Hochberg, 2000; Benjamini & Yekutieli, 2001). The smallest p-value is ranked one, next smallest is two, and the largest p-value has the rank of ten for ten tests, for example, then comparing each p-value to its Benjamni-Hochberg (BH) critical value. This critical value is calculated using the formula, (i/m)Q, where i is the rank, m is the total number of tests, and Q is the false discovery rate. The largest p-value, which is smaller than the BH critical value (*p*<(i/m)Q) are significant (Statistics How To, 2020). We used 5% false discovery rate although it is considered conservative. McDonald (2014) states that “all the p-values smaller that it are also significant, even the ones that are not less than their Benjamini-Hochberg critical value” (pp. 254-260).

To address the fourth research hypothesis, the percentages measuring participants' adherence for attending Internet and in person sessions were compared using proportion comparison for different total numbers using the formula,


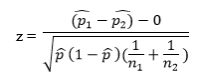


where p_1_ is the proportion of attendance for group sessions,

p_2_ is the proportion of attendance for Internet sessions,

p is the proportion of attendance for total sessions

n_1_ and n_2_ are the number of total attendance for group and internet sessions, respectively.

All statistical analyses used SPSS 26.0 and the significance level was set at .05.

## RESULTS

The results for demographic information included age, sex, ethnicity, marital status, affected side, and length of time since stroke are shown in Table 1. The results show no significant differences between Group 1 (higher functioning) and Group 2 (lower functioning). For months post stroke, both groups fit into the 24 to 36-month category post-stroke.

**Table 1.** Participant Characteristics

		Group 1 (Higher function)	Group 2 (Lower function)	Difference T or χ^2^ and (p)
Age		58.4 (11.4)	53.3 (16.9)	t=.574 (p=.571)
Ethnicity	White Black Hispanic	13 (86.7%) 2 (13.3%) 0	10 (76.9%) 2 (15.4%) 1 (7.7%)	χ^2^=1.255 (p=.534)
Sex	Male Female	9 (60%) 6 (40%)	5 (38.5%) 8 (61.5%)	χ^2^=1.292 (p=.256)
Marital Status	Married Single	8 (53.3%) 7 (46.7%)	11 (84.6%) 2 (15.4%)	χ^2^ = 3.125 (p=.077)
Months post-stroke	1 = < 12 2 = 12-24 3 = 24-36 4= 36-48 5 = 48 or >	3 (1.4)	3.23 (1.5)	t=.407 (p=.688)
Affected side	Left Right	8 (53.3%) 7 (48.7%)	10 (76.9%) 3 (23.1%)	χ^2^ =1.688 (p=.194)

Pretest comparison scores between Group 1 and Group 2 on functional outcome measures are detailed in Table 2. Since there are eight tests, using Benjamini-Hochburg (BH) method, we used (*i*/8) * .05 to calculate the critical value, and the original p-value was compared with the BH critical value (The last column in Table 2). The results show that Group 1 had significantly better scores at pretest compared to those of Group 2 on all outcome measures except the FIM self-care portion.

**Table 2.** Comparison of Pretest Scores for High and Low Functioning Groups

Outcome Measures	Pretest Mean (SD) Group 1 (High Functioning)	Pretest Mean (SD) Group 2 (Low Functioning)	Difference t (p)	BH critical value
WMFT (Time)	16.03 (10.2)	63.54 (23.9)	6.644 (p<.00001) *	.00625
WMFT (Function)	3.01 (.66)	2.0 (.60)	4.221 (p=.00013) *	.01875
WMFT (strength)	10.9 (8.1)	5.03 (4.3)	2.340 (p=.01360) *	.03750
Grip (kg)	8.3 (7.5)	4.03 (4.9)	1.820 (p=.04053) *	.04375
Hand to box (lbs.)				
FMA-UE	48.13 (10.3)	35.6 (13.5)	2.774 (p=.00505) *	.03125
MAL AOU	50.33 (26.8)	17.58 (13.7)	3.973 (p=.00025)*	.02500
MAL QOU	52.23 (22.7)	20.6 (14.9)	4.282 (p=.00011) *	.01250
FIM Self-care	36.67 (5.6)	33.08 (6.9)	1.487 (p=.07483)	.05000

* Significant against Benjamini-Hochberg critical value

Testing hypotheses 1-3, pre and posttest comparisons are shown in Table 3. Since we used 16 tests altogether, BH critical values were calculated using 16 tests (shown in the last column in Table 2). Both groups showed significant improvement for all outcome measures except for the timed WMFT and the grip strength sub-test of the WMFT for Group 1, and hand to box strength subtest for Group 2.

**Table 3.** Pre and Post Test

Group 1: High Functioning				
Outcome Measure	Pretest	Posttest	Difference t (p)	BH critical value
WMFT Time (seconds)	16.0 (10.2)	14.1 (13.0)	.613 (p=.27566)	.05000
WMFT (Function)	3.0 (.66)	3.62 (.86)	5.481 (p=.00004)*	.00625
WMFT (strength)				
Grip (kg)	10.9 (8.1)	12.9 (7.5)	1.620 (p =.06379)	.04375
Hand to box (lbs.)	8.3 (7.5)	10.6 (7.6)	2.718 (p=.00833)*	.034375
FMA-UE	48.1 (10.3)	51.8 (7.9)	2.859 (p=.00638)*	.03125
MAL AOU	50.3 (26.8)	72.6 (29.4)	5.760 (p=.00003) *	.003125
MAL QOU	52.3 (22.7)	70.8 (29.6)	4.493 (p=.00025) *	.01875
FIM Self-care	36.7 (5.6)	39.3 (6.2)	2.987 (p=.00489)*	.02500
**Group 2: Low Functioning**				
**Outcome Measure**	**Pretest**	**Posttest**	**Difference t (p)**	**BH critical value**
WMFT Time (seconds)	63.5 (23.9)	57.1 (27.1)	2.217(p=.02335)*	.03750
WMFT (Function)	2.0 (.6)	2.2 (.60)	3.001 (p=.00552)*	.02815
WMFT (strength)				
Grip (kg)	5.0 (4.3)	6.6 (4.8)	2.214 (p=.02334)*	.040625
Hand to box (lbs.)	4.0 (4.9)	4.6 (4.9)	.625 (p=.27184)	.046875
FMA-UE	36.6 (13.5)	42.2 (10.1)	4.614 (p=.00030)*	.01250
MAL AOU	17.6 (13.7)	38.4 (20.9)	4.495(p=.00036)*	.015625
MAL QOU	20.6 (14.9)	39.9 (21.3)	4.744 (p=.00024)*	.009375
FIM Self-care	33.1 (6.9)	37.8 (5.3)	3.566 (p=.00221)*	.021875

* Significant against Benjamini-Hochburg critical value.

Adherence for Internet session and in-person group sessions are detailed in Table 4. The results show that the adherence for Internet sessions was higher, (84.5%) than in-person group session (75.3%). Z score for the proportion difference was 2.65 (p=.004), showing Internet session attendance was significantly higher than that for group sessions.

**Table 4.** Session Adherence

	Total Number of Sessions	Number of Absences from Sessions	Percentage of Attendance
Group Sessions	182	45	75.3%
Internet Sessions	418	65	84.4%

## DISCUSSION

The addition of telehealth to a modified CIMT protocol is an innovative approach ensuring compliance, providing education for participants and families, and overcoming financial and transportation barriers. Based on our findings, both high and low functioning participants demonstrated significant improvements across a variety of assessments that measured functional use and quality of use in the hemiparetic UE. The protocol was adapted from the original design created by Taub et al. (1994), with an emphasis on retaining the three core components of CIMT including: intensive and graded use of affected limb, constraint of the unaffected limb, and the transfer package; use of the limb in daily activities.

The results showed significant improvements on both the amount of use and quality of use in functional participation measured by the Motor Activity Log (MAL) in a very similar manner for both higher and lower functioning groups. Significant improvements were also shown on the functional ability portion of the WMFT's functional ability scale for both groups. There were no significant differences on the timed WMFT among the higher functioning group. For the higher functioning group, it was observed during the posttest that they were more focused on the quality of their movements and their ability to achieve the desired actions on the WMFT as opposed to completing the functions with speed during the posttest sessions.

Finally, for the strength sub-tests of the WMFT, Group 1 demonstrated a positive change in hand to box performance and Group 2 demonstrated a positive change in grip strength. Many functional tasks were introduced during the Internet and group sessions involving grasp and release; it is possible that the lower functioning group benefitted from the increased attention to tasks involving grasp and their grip strength improved for these reasons. For the hand to box subtest, most of the participants in the higher functioning group demonstrated greater ability to raise the shoulder against resistance than the lower functioning group both at baseline and post intervention.

Results for the WMFT and MAL in this study were similar to Page and Levine's (2007) MAL and WMFT scores except for the WMFT timed portion for Group 1. Page and Levine utilized a webcam and video-conferencing system to deliver a 10-week CIMT training program. Additionally, the findings of this study were consistent in many areas with studies conducted by Taub et al. (2005), Lum et al. (2004), and Brennan et al. (2011). These studies explored the efficacy of conducting telerehabilitation using platform technology that specifically monitored repetitive task performance and promoted motor re-learning through shaping. Although our study did not have access to sophisticated technology platforms, the concepts of motor relearning and shaping were incorporated into the in-person and telehealth sessions. The results suggest that the concepts of motor re-learning and shaping using telehealth resulted in similar functional improvements, regardless of the varying systems.

It is important to note the delineation of the data related to the months post-stroke for the participants. The months post-stroke was in the 24-36-month range. Significant gains were still made in the categories of WMFT (functional ability scale), the FMA-UE, the MAL (AOU, QOU), and FIM self-care, aligning with the results found by Wolf et al. (2008) who studied the effect of CIMT on hemiparesis 1-2 years after the onset of stroke. These findings are consistent with the premise that individuals with chronic stroke suppress the use of the affected UE resulting in “learned non-use” and intensive training in the use of the affected limb results in cortical reorganization and functional improvements (Page et al., 2004).

Both groups significantly improved in FIM scores for self-care tasks.

In our study, the lower functioning group reported at baseline that they relied on a caregiver to do the task if it was too difficult. Improvements on the FIM, expressly for the lower functioning group, may be credited to caregiver education. Many caregivers attended our sessions and were able to witness the abilities and capacities of their significant others. It is possible that they may have learned how to be more encouraging with their significant others and step back from “doing the task” for their loved one.

Many studies do not show significant improvement for ADLs since individuals tend to compensate using the unaffected to perform daily tasks (Ploughman & Corbett, 2004). However, we speculate that in addition to caregiver education, our study participants became more actively engaged in ADLs because of the emphasis in our program on problem solving and learning new ways of performing tasks (Almhdawi et al., 2014; Chan et al., 2006; Sabari et al., 2014). This behavior was encouraged and threaded throughout the sessions. We do not know what instructions were discussed with study participants in other studies; however, we believe not only emphasizing repetition but also introducing a new mind-set should be part of CIMT training. Improving functional status in ADLs provides encouragement and confidence. A decline in ADL abilities has been associated with depression (Tomita et al., 2004), whereas; engagement in either telehealth or monitored home programs has been shown to decrease depression in individuals post stroke (Linder et al., 2015).

Finally, although this study combined traditional in-person group therapy and home-based individual therapy utilizing the Internet, when we compared attendance rates, the Internet sessions were attended with greater consistency compared to the group sessions. The telehealth approach allowed for increased flexibility which facilitated adherence to the program. By implementing video-conferencing sessions, the participants were able to engage in therapy sessions from the comfort of their own homes. This largely eliminated the need to travel as frequently as traditional therapy requires, making this an accessible way to deliver services to individuals in the future. This was a benefit for our participants since this program was conducted during the winter months in a Northeastern city. Participants were encouraged to engage in meaningful activities in their natural home environments. However, we do not conclude that in-person group therapy can be eliminated.

Telehealth programs have limitations including the fact that therapists are unable to provide hands on guidance and must rely on verbal instruction and visual demonstration for the participant to understand the exercise or activity. In-person group sessions once a week allowed the opportunity to assess participants' progress accurately, provide tactile guidance, and make appropriate recommendations for future sessions. Therefore, we believe the combination of Internet and in-person sessions provided the best of both worlds. The two Internet sessions per week lessened the burden on participants and caregivers for transportation; meanwhile, the once per week in-person sessions provided the hands-on therapeutic interaction between the therapists and participants. An additional component was the opportunity for participants to engage in social interaction, an important component in recovery. This opportunity may have provided further encouragement for them to continue home-based telehealth sessions, providing a notion that they are not alone in doing it. Comparison of the effectiveness of this combined approach and the cost of delivery are encouraged for future studies.

A major limitation for most CIMT studies pertains to the strict inclusion criteria, which is derived and modified from the traditional CIMT approach. The strict inclusion criteria result in difficulty with recruitment of participants, and it excludes willing participants who desire to regain function of their affected UE; however, studies have been conducted with participants who do not meet the criteria for hand function. Taub et al. (1999) report improvements in real world function for participants who are lower functioning. This study included participants who did not meet the criteria for CIMT studies and improvements in UE function was achieved.

The American Occupational Therapy Association's (AOTA) position on telehealth sets guidelines for the delivery of occupational therapy services using telehealth to promote participation (AOTA, 2018). The occupational therapy practitioner must adhere to occupational therapy practice guidelines and ethical practice whether in-person or using web-based technologies to provide services. Among considerations when implementing a telehealth program are individuals who are not familiar with technology and who may struggle with Internet access and become frustrated (Jacobs et al., 2015). However, obstacles such as these may be overcome with participation from family members and/or significant others. We found that many times family members were willing to assist participants and set them up with the technology and join in with them during the session. We observed that spousal support was valuable, especially for our lower functioning group, and they benefitted from the interactions with their spouses/significant others throughout our sessions. We made it a point to include spouses and significant others in our sessions and encouraged their participation. Spouses and significant others were encouraged to provide thoughtful and creative problem-solving opportunities for the participants and to assist them with the technologies that were new to them or unfamiliar. Therefore, we suggest that a family-centered approach is important for post stroke therapy and telehealth is an integral element for the approach.

## CONCLUSION

By combining mCIMT with telehealth and traditional group rehabilitation, our study demonstrated statistically significant improvements in function as well as the participants' perceived use of hemiparetic upper extremity for both higher and lower functioning participants. Future studies should utilize a larger sample size to improve generalizability and the addition of a control group for a higher level of evidence. In addition, follow up assessment after a three-month period would determine if the gains made in the program were sustained. We further recommend that future researchers consider the use of group therapy telehealth sessions to improve participation, motivation, socialization, and group comradery.
